# Aerobic Vaginal Microflora in Gestational and Non-Gestational Bitches (*Canis lupus familiaris*)

**DOI:** 10.3390/ani14101501

**Published:** 2024-05-18

**Authors:** Raul Alexandru Pop, Iosif Vasiu, Gabriele Meroni, Piera Anna Martino, Roman Dąbrowski, Asta Tvarijonaviciute, Iosif Nicodim Fiţ

**Affiliations:** 1 Department of Obstetrics and Gynecology, Faculty of Veterinary Medicine, University of Agricultural Science and Veterinary Medicine, 400372 Cluj-Napoca, Romania; raul.pop@usamvcluj.ro; 2 Department and Clinic of Infectious Disease, Faculty of Veterinary Medicine, University of Agricultural Science and Veterinary Medicine, 400372 Cluj-Napoca, Romania; iosif.vasiu@usamvcluj.ro; 3 One Health Unit, Department of Biomedical, Surgical and Dental Sciences, University of Milan, 20133 Milan, Italy; piera.martino@unimi.it; 4 Department and Clinic of Animal Reproduction, Faculty of Veterinary Medicine, University of Life Sciences, 20-612 Lublin, Poland; roman.dabrowski@up.lublin.pl; 5 Interdisciplinary Laboratory of Clinical Analysis (Interlab-UMU), Regional Campus of International Excellence “Mare Nostrum”, University of Murcia, 30100 Murcia, Spain; asta@um.es; 6 Department of Microbiology, Mycology, and Immunology, Faculty of Veterinary Medicine, University of Agricultural Science and Veterinary Medicine, 400372 Cluj-Napoca, Romania; nfit@usamvcluj.ro

**Keywords:** bitch, milk, vaginal microbiology

## Abstract

**Simple Summary:**

Commensal bacteria in the vaginal canal are vital to reproductive health. Infertility or severe urinary tract infections may result from vaginal dysbiosis. This research examined the aerobic bacterial flora in vaginal samples from bitches during antepartum, postpartum, and *Lactatio sine graviditate*. The 100 vaginal samples analyzed yielded 82% positive microbiological results and 18% negative. Microbiologic profile revealed 17 genera. Micrococcaceae, Staphylococcaceae, Morganellaceae, Bacillaceae, and Rhizobiaceae were the most isolated bacteria. The first strains of *Agrobacterium radiobacter*, *Ochrobactrum anthropi*, *Chromobacterium violaceum*, *Burkholderia mallei*, *Bacillus pumllus*, and *Staphylococcus xylosus* were recovered from vaginal secretions. Microbiological studies show that breastfeeding bitches’ vaginal discharge is variable and may be impacted by coitus, sampling season, age, and reproductive status.

**Abstract:**

The vaginal tract comprises commensal microorganisms, which play an essential role in the health of the reproductive tract. Any dysbiosis in the vaginal microenvironment may lead to severe urinary tract infections or even infertility. This study aimed to evaluate the aerobic bacterial flora isolated from vaginal samples from 100 lactating bitches in the antepartum period (*n* = 3), postpartum period (*n* = 80), and with *Lactatio sine graviditate* (*n* = 17). Before vaginal swabs, all the bitches went through a gynecology consult, along with milk and blood sampling. Standard microbiological techniques were used for bacterial isolation. Among the 100 vaginal samples analyzed, 82% had a positive microbiological outcome, while 18% were negative. The microbiologic profile listed 17 different genera. The main isolated bacterial families were Micrococcaceae, Staphylococcaceae, Morganellaceae, Bacillaceae, and Rhizobiaceae. At the same time, strains like *Agrobacterium radiobacter*, *Ochrobactrum anthropi*, *Chromobacterium violaceum*, *Burkholderia mallei*, *Bacillus pumllus*, or *Staphylococcus xylosus* were isolated for the first time from the vaginal secretion of lactating bitches. The microbiological data demonstrates that lactating bitches’ vaginal discharge is heterogeneous and may be affected by coitus, sampling season, age, and reproductive status.

## 1. Introduction

The microbiota and the microbiome consist of all the conjoint microorganisms and genes that live within the hosts, modulating the health or disease processes. Alterations in microbiota composition are known as dysbiosis [1]. Understanding the vaginal microbiota is key to effectively preventing urinary tract infections (UTI) [2].

The presence of commensal microorganisms in the reproductive tract has a role in establishing a fundamental and strong immune state within this particular habitat. One plausible major function of the microbiota in the reproductive tract might be the alteration or limitation of certain constituents within other bacteria in different body regions [3]. Specific phyla have been identified among the different species and within distinct areas of the female reproductive tract, including the vagina; however, the microbiota’s function in the reproductive tract is not yet fully understood [3].

Typically, canine vaginal flora comprises a dynamic bacterial population, encompassing both aerobic and anaerobic microorganisms. The bacterial species that are frequently encountered in the canine vaginal tract consist of *Staphylococcus pseudintermedius*, *Streptococcus canis*, *Enterococcus* spp., and *Mycoplasma* spp. [4]. It is worth noting that lactobacilli, which make up the majority of the vaginal microbiota in humans, are rarely isolated from the vaginal tracts of canines. A greater vaginal pH range (5.0 to 8.1) may be a result of the absence of lactobacilli in canines as opposed to humans. Additionally, even in healthy individuals, the presence of bacteria in the vaginal tract of canines, whether unbound or within epithelial cells, is a common occurrence [2].

The description of the microbiota in the reproductive tract has enabled the detection of subtle changes that may not manifest clinically but might have important clinical significance. Therefore, obtaining a more comprehensive picture of the bacterial microbiome inside the female reproductive system is advantageous, since this knowledge may assist in treating genital tract infections and reproductive failures [3].

Variation in the vaginal microbiota is observed at various stages of the estrous cycle. *S. canis* is more prevalent during proestrus, whereas the presence of *Enterococcus* spp. is more frequently observed in dogs with genital tract infections. *Staphylococcus* spp. and *Streptococcus* spp. have the potential to serve as protective agents against more harmful pathogens through mechanisms such as nutrient competition and epithelial cell receptor adhesion interference [2,5]. Studies have shown that a varied bacterial community (including aerobic and anaerobic microbes and opportunistic pathogens) is present in the vaginas of 50% to 100% of clinically healthy dogs [2]. The typical vaginal microbiota is thought to safeguard the genito-urinary tract against harmful organisms, and diseases in this area are often associated with alterations in the vaginal microflora. Research indicates that bacterial species found in female dogs with reproductive disorders are not considerably different, indicating that infections in the reproductive system may be caused by an excessive expansion of the usual local microbial population [6]. So far, various publications have researched the vaginal microbiota in different cycle stages, including anestrus or even for spayed (ovariohysterectomy; OHE) bitches [7,8,9,10]. Moreover, the microbiota of the genital tract (i.e., vagina, cervical, and uterus) of different reproductive cycles [5,11] and between healthy and ill bitches with various urogenital problems (i.e., infertility UTI and neonatal mortality) were also assessed [12,13,14,15,16].

As information related to the bitch vaginal tract microbiome is quite heterogenous, this study aims to evaluate the prevalence of aerobic bacteria in the vagina according to the lactation period, the type of coitus, seasonality of sampling, age, and reproductive status of gestational and non-gestational bitches.

## 2. Materials and Methods

### 2.1. Animals

One hundred lactating bitches between 10 and 168 months of age (45.29 ± 27.62 SD) and weighing between 3 and 65 kg (28.51 ± 27.47 SD) were included in this research study. The bitches were first examined at the Department, and Clinic of Reproduction, Obstetrics and Gynaecology, University of Agricultural Sciences and Veterinary Medicine, Cluj-Napoca, Romania, between January 2013 and December 2014.

In total, 52 (52%) were multiparous, 32 (32%) were primiparous, and 10 (10%) were intact bitches; for the remaining 6, no data were available. Furthermore, 3 bitches (3%) were in the antepartum period (24/48 h before parturition or C-section), 80 (80%) were postpartum (1st week to 6th week after parturition), and 17 (17%) were diagnosed with *Lactatio sine graviditate* (LSG; 45–60 days after normal estrus when progesterone levels begin to decrease). Regarding mating, 13 bitches (13%) were artificially inseminated, 55 (55%) were naturally controlled reproduced, and 12 (12%) were accidentally reproduced, while, for 20 females, no data were available. Furthermore, 29 samples were collected during spring (March–May), 26 were collected during summer (June–August), 25 were collected during autumn (September–November), and 20 were collected during winter (December–February).

The dogs included in the current study belong to 30 different breeds (including cross breed), namely German Shepherd (*n* = 21), Cane Corso (*n* = 9), mongrels (*n* = 8), Rottweiler (*n* = 7), Caucasian Shepherd Dog (*n* = 6), Siberian Husky (=5), Bichon (=4), Dobermann (=4), Yorkshire Terrier (=4), American Staffordshire Terrier (=2), American Bulldog (=2), Beagle (=2), Bucovina Shepherd Dog (=2), Cocker Spaniel (=2), Dachshund (=2), English Bulldog (=2), French Bulldog (=2), German Shorthaired Pointer (=2), Miniature Schnauzer (=2), Pekingese (=2), Saint Bernard (=2), and one Basset Hound, Belgian Shepherd, Boxer, Central Asian Shepherd Dog, Golden Retriever, Labrador retriever, Neapolitan Mastiff, Shih Tzu, and Vizsla from each breed.

The clinical evaluation and diagnostic methodology, including hematological changes, milk and serum acute phase protein levels (APPs), milk cytology evaluation, and milk microbiome results, used to assess the health status of the bitches included in the current study were published by the authors of this research in several other papers [17,18,19]. In total, 40 bitches were healthy; 3 were diagnosed with mammary congestion, 12 with galactostasis, 17 with subclinical mastitis, 12 with acute mastitis, and 1 with gangrenous mastitis, while the other 15, due to lack of data or preliminary laboratory testing, had no definitive diagnostic.

### 2.2. Sample Collection and Microbiological Analysis

After thoroughly disinfecting the vulvar region with warm water, soap, and ethanol (70% *v*/*v*), one vaginal swab was taken from each bitch using a sterile bacteriological swab with transport medium (Stuart medium) [20].

For the microbiological assays, standard microbial methods were used. Thus, bacteriological samples were incubated for 24 h at 370 °C in brain–heart infusion broth (Oxoid, Ltd., Basingstoke, Hampshire, UK). Furthermore, for Staphylococcal growth, the culture was inoculated on Chapman medium agar (Oxoid, Ltd., Basingstoke, Hampshire, UK). Streptococci were grown on blood agar (Oxoid, Ltd., Basingstoke, Hampshire, UK), while Enterobacteriaceae were grown on McConkey medium agar (Oxoid, Ltd., Basingstoke, Hampshire, UK). Finally, the isolates were identified using the Vitek2 identification system (BioMérieux, l’Étoile, France), following the manufacturer’s guidelines.

### 2.3. Statistical Analysis

The anamnestic data, clinical outcome, and microbiological findings were recorded and saved on an Excel spreadsheet. The data were analyzed using GraphPad Prism version 8.0.0 for Windows, a software developed by GraphPad Software from San Diego, CA, USA. A chi-squared test was used to evaluate the statistical disparities in prevalence. A *p*-value below the threshold of 0.05 was deemed statistically significant.

## 3. Results

### 3.1. Microbiology Results

From the 100 vaginal samples collected, 18% (18/100) were negative for microbiological culturing, while 82% (82/100) were positive, out of which 12.2% (10/82) were from bitches with LSG. From the isolated microbial samples, 63.55% (68/107) were Gram-positive bacteria (G+) and 36.55% (39/107) were Gram-negative (G−). Furthermore, up to 17 genera (among which 7 G+ and 10 G−) were identified as follows: *Staphylococcus* (79.4%), *Proteus* (28.2%), *Agrobacterium* (23.1%), *Pseudomonas* (12.8%), and *Bacillus* (11.8%) as the predominant vaginal strains (Table 1).

Co-isolations of different bacteria were observed in 27% (27/100) animals. *Staphylococcus* and *Bacillus* were detected in 4% of cases, followed by *Agrobacterium* and *Pseudomonas* (2%) and *Staphylococcus* and *Agrobacterium* (2%) (Table S1). 

### 3.2. Bacterial Distribution According to Lactation Period

When it came to the population during the antepartum period, the family Staphylococcaceae had the highest prevalence of isolation, with 50 percent of them. This was followed by the Streptococacceae and Burkholderiaceae, both of which represented 25% of the cases. The Bacillaceae family was responsible for 23.1% of the cases, making it the second-most common family in terms of frequency. After this, the Morganellaceae, Pseudomonadaceae, and Micrococcaceae families each had a predominance of 7.7%. This was the next family in the classification. In the case of females with LSG, the Staphylococcaceae family had the greatest prevalence, reaching 53.8% of the total. According to Table 2, the Staphylococcaceae family had the highest frequency of isolation during the postpartum period, with 48.4% of cases. This was followed by the Morganellaceae family, which had 10.8% of cases, and the Rhizobiaceae family, which had 9.7% of cases. 

The comparison of the antepartum period with the postpartum period revealed a significant difference for the families of Brucellaceae, Aerococcaceae, Enterococcaceae, Micrococcaceae, Aeromonadaceae, and Weekesellaceae (Table 2).

### 3.3. Bacterial Distribution According to Reproductive Status

As reported, 44.4% of intact females were positive for Staphylococcaceae, the family with the highest prevalence of isolation. The subsequent most prevalent family, with 33.3%, was Bacillaceae, followed by Enterobacteriaceae and Morganellaceae with 11.1% each. A proportion of 50.9% of the cases involving multiparous female were attributed to the Staphylococcaceae family, which had the highest incidence rate. With a prevalence of 12.7%, this was succeeded by the Rhizobiaceae family. The incidence rate was 5.5% for each entry in the Bacillaceae, Streptococcaceae, Morganellaceae, and Pseudomonadaceae families. The most prevalent taxa in primiparous female canines were Staphylococcaceae and Micrococcaceae, which accounted for 33.9% of all cases, respectively. An incidence rate of 11.9% was observed in the Morganellaceae family, which followed closely. Table 3 displays that the occurrence rate was identical for each of the following genera: Rhizobiaceae, Bacillaceae, Streptococcaceae, Neisseraceae, and Enterobacteriaceae. In addition, when comparing intact bitches to both multiparous and primiparous bitches, a statistically significant distinction was observed in the Bacilaceae family. Furthermore, by comparing multiparous and primiparous bitches within the Micrococcaceae family, a statistically significant distinction was observed (Table 3).

### 3.4. Bacterial Distribution According to the Type of Coitus

The Staphylococcaceae family exhibited the highest prevalence of isolation (37.5%) among bitches that underwent artificial insemination. Subsequently, the Rhizobiaceae (18.8%) and Morganellaceae (12.5%) families were observed. The Staphylococcaceae family exhibited the highest incidence among females with controlled natural reproduction (60.7%). This was followed by Rhizobiaceae (8.9%) and Morganellaceae (7.1%). Among the subjects, the following families, Staphylococcaceae, Micrococcaceae, and Morganelaceae, demonstrated the highest prevalence of uncontrolled natural reproduction, with each family contributing 19.0%. This was closely followed by the Enterobacteriaceae family at 14.3% and the Bacillaceae family at 9.5%. 

Furthermore, a distinction that was statistically significant was observed exclusively within the Staphylococcaceae family when contrasting bitches whose reproduction was naturally controlled with those whose reproduction was naturally uncontrolled (Table 4).

### 3.5. Bacterial Distribution According to Sampling Season

A significant proportion of the isolated bacteria (48.5%) belong to Staphylococcaceae, which was the highest prevalence detected during the autumn season. The subsequent families to be isolated were Pseudomonadaceae and Rhizobiaceae, with respective prevalences of 12.9% and 22.6%. With 69.2% of all observed cases occurring during the spring, the Staphylococcaceae family demonstrated the highest prevalence of isolation. A proportion of 15.4% of the isolates were attributed to the Enterobacteriaceae family. A frequency of isolation of 7.7% was observed for both the Bacillaceae and Morganellaceae families. Summertime saw the maximum frequency of Staphylococcaceae isolations, which accounted for 50 percent of all cases observed. The subsequent families to contribute to the overall prevalence were Morganellaceae, Bacillaceae, and Streptococcaceae, each with a 12.5% share. Comparatively, the prevalence of the Enterobacteriaceae family was 4.2%. The winter exhibited the highest frequency of occurrence among the following families: Staphylococcaceae (25.7%), Morganellaceae (14.3%), and Micrococcaceae (31.4%).

Further, an abundance disparity of the Rhizobiaceae family was observed to be statistically significant between autumn, spring, and summer. Additionally, when the Micrococcacea family’s autumn, spring, and summer seasons were compared to the winter season, a substantial statistical discrepancy was observed. In conclusion, an abundance variation of the Staphylococcacea family that was statistically significant was observed during the transition from spring to winter (Table 5).

### 3.6. Bacterial Distribution According to Age and Reproductive Status

The Bacillaceae family exhibits the highest prevalence among intact females under 2 years (42.9%). The Staphylococcaceae family follows with 28.6% of cases, while the Aerococcaceae and Enterobacteriaceae families each contribute 14.3% of cases. The highest occurrence rate (50%) was observed in the Staphylococcaceae family among multiparous females older than two years; the Rhizobiaceae family followed with a rate of 13.5%. The families Morganellaceae, Bacillaceae, and Streptococcaceae each exhibited an incidence rate of 5.8%. The prevalence of the Staphylococcaceae family was found to be highest among primiparous bitches younger than two years at 45%. The Morganellaceae family ranked second with 15%, while the Rhizobiaceae family accounted for 10%. In contrast, intact bitches that were two years of age or older exhibited the greatest prevalence of the Micrococcaceae and Staphylococcaceae families, each accounting for 36.7%. The prevalence of the Morganellaceae family was 13.3%, whereas the prevalence of each of the Bacillaceae, Streptococcaceae, Neisseriaceae, and Enterobacteriaceae families was 3.3% (refer to Table 6 for details). However, no differences that were statistically significant were observed between the compared groups. 

## 4. Discussion

In the current research, 17 bacterial genera were isolated (i.e., 7 G+ and 10 G−). Most G+ genera isolates consisted of *Staphylococcus* and *Bacillus*, while the G- genera isolates comprised *Proteus*, *Agrobacterium,* and *Pseudomonas* (Table 1). Associations between G+ and G− bacteria from the same vaginal sample, like *Staphylococcus* spp. and *Escherichia coli* or *Staphylococcus* spp. and *Agrobacterium radiobacter*, were frequently encountered (Table S1).

The cranial vagina of mammals harbors a thriving microbial ecosystem [5] dominated by Proteobacteria, Bacteroidota, and Firmicutes phyla [3,5]. Interestingly, the results of our study are consistent with the current data from the literature.

However, about 60% of the species in the vagina belong to the *Hydrotalea*, *Ralstonia*, *Fusobacterium*, or the *Mycoplasma* and *Streptococcus* genera [2,5,10,20,21]. Nevertheless, concerning these reported data, our study identified only strains from the *Burkholderia* genera (i.e., *Burkholderia cepacia*). This result should be carefully interpreted since molecular screening for Mycoplasma and Chlamydia species were not performed.

Furthermore, 82% of our tested vaginal samples were positive for the microbiological culture. These findings are consistent with other reports, where about 71–79% of all the tested bitches had a positive vaginal microbiological culture result [7]. 

The literature reports the presence of many bacterial isolates like *Actinomyces coleocanis*, *Arcanobacterium pyogenes*, *Corynebacterium genitalium*, *Enterobacter aerogenes*, *Enterococcus avium*, *E*. *canintestini*, *E*. *cloacae*, *E*. *durans*, *E*. *faecalis*, *Erwinia herbicola*, *Klebsiella pneumoniae*, *Koserella trabulsii*, *Obesumbacterium proteus*, *Pasteurella multocida*, *Proteus mirabilis*, *Pseudomonas aeruginosa*, *Serratia rubidea*, *Sphingomonas paucimobilis*, *Staphylococcus aureus*, or even *Candida* spp. [7,8,9,11,14,15,22,23].

To the author’s knowledge, this is the first study where *A. radiobacter*, *Ochrobactrum anthropi*, *Chromobacterium violaceum, Burkholderia mallei*, *Bacillus pumilus*, *Streptococcus anginosus*, *Streptococcus sanguinis*, or *S. xylosus* have been isolated from the bitch vaginal secretion (Table S1). Moreover, literature research shows that the mammalian vagina contains a site-specific microbiota that holds a regulator and/or a drive essential role in many physiological and pathological processes in genital and reproductive health [24]. 

However, it is also shown that the vagina is much higher in richness but lower in diversity than the endometrium [5,21]. Interestingly, in the present study, the diversity of vaginal strains was richest in the postpartum group compared to the LSG group. The lack of sexual activity for LSG bitches could explain the difference in the population diversity, while, for the postpartum bitches, the opening of the cervix and the puppy passages certainly facilitated the increased diversity of the isolated strains.

Nevertheless, there is significant animal-to-animal variation in the vaginal microbiota [5], geographical regions influencing the presence of acid-producing bacteria (LAB) [25], while stray dogs may be potential reservoirs of pathogenic antimicrobial-resistant microorganisms [23]. Furthermore, age, breed, sex, lifestyle (i.e., urban or rural), or diet influences the core microbiota for different body sites (including the urogenital tract) [1]. 

Thus, the prevalence of the vaginal microbiota was affected in the current study by the lactation period (antepartum, postpartum, or LSG), reproductive status (intact, primiparous, or multiparous), type of coitus (naturally controlled, naturally uncontrolled, or artificial insemination), and sampling season (winter, spring, summer, or autumn). The prevalence of the bitch vaginal microbiota has never been assessed using these criteria, as far as the authors are aware.

The vaginal microbiota of bitches in different reproductive phases exhibits a significant level of diversity and variability [10,15,23,25]. Nevertheless, between intact and OHE bitches, the vaginal microbiome heterogenicity is reduced [10]. However, in one study, the most present genera in OHE bitches were *Photobacterium* (14.03%), *Staphylococcus* (11.19%), *Mycoplasma* (7.64%), and *Salmonella* (11.19%), followed by the Enterobacteriaceae (10.01%) family. In contrast, for anestrus bitches, the most encountered genera were *Mycoplasma* (13.90%), *Salmonella* (7.60%), and *Staphylococcus* (6.80%), followed by the Pasteurellaceae (7.84%) and the Enterobacteriaceae (6.27%) families [10]. 

However, a variety in quantity and type of bacteria between different estrous cycle stages [2] is noticed, especially for females in estrus, where the richness in diversity of the vaginal strains is at the top [5,21]. For OHE or infertile bitches, *E. coli*, *S. pseudintermedius*, and *S. canis* are the predominant strains identified [10,15,23,25].

Our findings are interesting in that, for artificially inseminated, naturally controlled, and uncontrolled coitus bitches, the levels of diversity and variability were similar; for primiparous and multiparous bitches compared to intact ones and for the autumn and winter seasons when compared to spring and summer, the levels of diversity and variability were higher.

Acid-producing bacteria are vaginal residents in the bitch [7,26], with only 3% of the isolates belonging to the *Lactobacillus* genus, with the majority of isolates corresponding to *Lactococcus* spp., followed by *Lactobacillus* spp., *Pediococcus acidilactici*, *Lactiplantibacillus plantarum*, or *Lactococcus lactis* [26]. Interestingly, no such LABs were isolated in our study due to the use of nonspecific microbiological media.However, in the vaginal vault of spayed bitches, LABs are not predominant [14]. 

No significant difference is found between spayed bitches with rUTI and healthy ones. While *E. coli* and *S. pseudintermedius* are the most often isolated organisms from the vaginal tract of bitches (both healthy and with rUTI), *E. coli* tends to be more common in females with rUTI. Moreover, *Enterococcus canintestini* was isolated from both healthy and rUTI bitches [25]. There was comparable prevalence of common vaginal infections among healthy bitches and those who had UTI. Consequently, the incidence of *E. coli* is 50% among ill and 42% among healthy bitches, while *S. canis* and *S. pseudintermedius* have prevalence rates of 30% and 38%, respectively [2]. Furthermore, in the proestrus phase, for healthy bitches, an increase in *Enterococcus* spp. and a decrease in the *E. coli* population is noted, with *S. canis* being much more common than *Enterococcus* spp. [2]. 

Some reports suggest that the presence of *Streptococcus* spp., *Lactobacillus* spp., or *Enterococcus* spp., including *E. canintestini*, during proestrus reduces the genital incidence of infections, highlighting a possible protective role for these strains through competitive and adhesion mechanisms for nutrients and the cells’ surface or having potential antimicrobial properties, such as the production of lactic acid, bacteriocin, and hydrogen peroxide, or due to blood irrigation of the vagina during proestrus detrimental to other possible pathogens [2,7,12,25].

Performing cultures of the vaginal canal will often yield bacterial growth, making it difficult to understand whether therapy is necessary. The process of cultivating bacteria from vaginal swab samples taken from female dogs that do not show any indications of genital illness has limited usefulness. Prior to it, clinical and cytological exams of the vaginal epithelium should always be conducted [2]. The authors of this research strongly discourage the use of unnecessary or prophylactic antibiotic treatments, as misuse of antimicrobials favors the selection of Methicillin-resistant *Staphylococcus pseudintermedius* (MRSP) strains in healthy dogs and their persistence over time [27].

However, using a broad-spectrum antibiotic doubled by a susceptibility test is mandatory whenever necessary. Noteworthy, a microbiological evaluation of vaginal samples may be used in bitches with a history of fetal mortality. It could be used for selectively treating such females before parturition, especially in cases where β-hemolytic *Streptococcus* is isolated [13]. 

The presence of phagocytosis and neutrophils on vaginal smears, often indicative of infections, are part of the normal physiological processes observed at the vaginal mucosal surface in bitches. Furthermore, bitches harboring vaginal isolates exhibit a greater fertility rate and produce more robust and healthier offspring compared to bitches without any cultured isolates. These processes are connected with a greater fertility rate [7]. 

Finally, there are also reports which show that the same isolates (i.e., *S. pseudintermedius*, *E. coli*, or *S. canis*) are cultured from both the vagina and the milk secretions during antepartum and postpartum [16], raising the question of whether or not vaginal bacterial strains are responsible for mammary gland infections, as bacterial translocation mechanisms are acknowledged [12,28,29].

The relatively low number of samples and the lack of molecular identification of isolates represent the main limitations of the current study. Furthermore, future research should focus on anaerobic bacterial cultures in periparturient bitches, to enhance the knowledge variability of bitch vaginal heterogenicity, and the possibility of bacterial translocation from the vagina or myometrium to the lactating mammary glands as a cause for the onset of mastitis.

## 5. Conclusions

The prevalence of the aerobic bacterial vaginal microbiome in lactating bitches can be influenced by the lactation period, the reproductive status, the type of coitus, or seasonality; however, it is not affected by age compared to the reproduction phase. 

Except for the winter season, where the Micrococcaceae family had the highest prevalence, all the other groups recorded the highest prevalence for the Staphylococcaceae family, followed by the Morganellaceae, Bacillaceae, or the Rhizobiaceae families.

## Figures and Tables

**Table 1:** Main vaginal bacterial genera found from microbiological analysis of vaginal swabs.

G+	N	Prevalence (%)	G−	N	Prevalence (%)
*Aerococcus*	1	1.5	*Aeromonas*	1	2.6
*Bacillus*	8	11.8	*Agrobacterium*	9	23.1
*Enterococcus*	1	1.5	*Burkholderia*	3	7.7
*Micrococcus*	1	1.5	*Chromobacterium*	2	5.1
*Rothia*	1	1.5	*Chryseobacterium*	1	2.6
*Staphylococcus*	54	79.4	*Escherichia*	3	7.7
*Streptococcus*	2	2.9	*Klebsiella*	3	7.7
			*Ochrobactrum*	1	2.6
			*Proteus*	11	28.2
			*Pseudomonas*	5	12.8
Total	68	100		39	100

**Table 2:** Relative abundance (%) of bacteria (family level) according to the lactation period.

Bacterial Families	AP (*n* = 3)	LSG (*n* = 13)	PP (*n* = 93)	AP vs. LSG	LSG vs. PP	AP vs. PP
Brucellaceae	0	0	1.1	ns	ns	0.0204
Rhizobiaceae	0	0	9.7	ns	ns	ns
Aerococcaceae	0	0	1.1	ns	ns	0.0204
Bacillaceae	0	23.1	5.4	ns	ns	ns
Enterococcaceae	0	0	1.1	ns	ns	0.0204
Micrococcaceae	0	7.7	1.1	ns	ns	0.0204
Staphylococcaceae	50	53.8	48.4	ns	ns	ns
Streptococcaceae	25	0	4.3	ns	ns	ns
Burkholderiaceae	25	0	2.2	ns	ns	ns
Neisseraceae	0	0	2.2	ns	ns	ns
Aeromonadaceae	0	0	1.1	ns	ns	0.0204
Enterobacteriaceae	0	0	6.5	ns	ns	ns
Morganellaceae	0	7.7	10.8	ns	ns	ns
Pseudomonadaceae	0	7.7	4.3	ns	ns	ns
Weeksellaceae	0	0	1.1	ns	ns	0.0204

Abbreviations: PP = *postpartum*; LSG = *Lactatio sine graviditate*; AP = *antepartum*; ns = not significant.

**Table 3:** Relative abundance (%) of bacteria (family level) according to reproductive status.

Bacterial Families	I (*n* = 9)	M (*n* = 55)	P (*n* = 59)	I vs. M	M vs. P	I vs. P
Brucellaceae	0	1.8	0	ns	ns	ns
Rhizobiaceae	0	12.7	3.4	ns	ns	ns
Aerococcaceae	0	0	0	ns	ns	ns
Bacillaceae	33.3	5.5	3.4	0.032	ns	0.0117
Enterococcaceae	0	0	0	ns	ns	ns
Micrococcaceae	0	1.8	33.9	ns	<0.0001	ns
Staphylococcaceae	44.4	50.9	33.9	ns	ns	ns
Streptococcaceae	0	5.5	3.4	ns	ns	ns
Burkholderiaceae	0	3.6	1.7	ns	ns	ns
Neisseraceae	0	0	3.4	ns	ns	ns
Aeromonadaceae	0	1.8	0	ns	ns	ns
Enterobacteriaceae	11.1	3.6	3.4	ns	ns	ns
Morganellaceae	11.1	5.5	11.9	ns	ns	ns
Pseudomonadaceae	0	5.5	1.7	ns	ns	ns
Weeksellaceae	0	1.8	0	ns	ns	ns

Abbreviations: I = intact; M = multiparous; P = primiparous; ns = not significant.

**Table 4:** Relative abundance (%) of bacteria (family level) according to the type of coitus.

Bacterial Families	A (*n* = 16)	Nc (*n* = 56)	Nu (*n* = 21)	A vs. Nc	Nc vs. Nu	A vs. Nu
Brucellaceae	6.3	0	0	ns	ns	ns
Rhizobiaceae	18.8	8.9	4.8	ns	ns	ns
Aerococcaceae	0	0	0	ns	ns	ns
Bacillaceae	0	3.6	9.5	ns	ns	ns
Enterococcaceae	0	0	4.8	ns	ns	ns
Micrococcaceae	6.3	0	19.0	ns	ns	ns
Staphylococcaceae	37.5	60.7	19.0	ns	0.0027	ns
Streptococcaceae	6.3	3.6	4.8	ns	ns	ns
Burkholderiaceae	0	3.6	4.8	ns	ns	ns
Neisseraceae	6.3	1.8	0	ns	ns	ns
Aeromonadaceae	0	1.8	0	ns	ns	ns
Enterobacteriaceae	0	1.8	14.3	ns	ns	ns
Morganellaceae	12.5	7.1	19.0	ns	ns	ns
Pseudomonadaceae	6.3	5.4	0	ns	ns	ns
Weeksellaceae	0	1.8	0	ns	ns	ns

Abbreviations: A = artificial; Nc = naturally controlled; Nu = naturally uncontrolled; ns = not significant.

**Table 5:** Relative abundance (%) of bacteria (family level) according to the sampling season.

Bacterial Families	A (*n* = 31)	Sp (*n* = 26)	Sm (*n* = 24)	W (*n* = 35)	A vs. Sp	A vs. S	A vs. W	Sp vs. S	Sp vs. W	S vs. W
Brucellaceae	3.2	0	0	0	ns	ns	ns	ns	ns	ns
Rhizobiaceae	22.6	0	0	5.7	0.0291	0.0372	ns	ns	ns	ns
Aerococcaceae	0	0	0	2.9	ns	ns	ns	ns	ns	ns
Bacillaceae	0	7.7	12.5	8.6	ns	ns	ns	ns	ns	ns
Enterococcaceae	0	0	0	0	ns	ns	ns	ns	ns	ns
Micrococcaceae	0	0	0	31.4	ns	ns	0.0020	ns	0.0048	0.0068
Staphylococcaceae	48.4	69.2	50	25.7	ns	ns	ns	ns	0.0018	ns
Streptococcaceae	3.2	0	12.5	2.9	ns	ns	ns	ns	ns	ns
Burkholderiaceae	3.2	0	8.3	0	ns	ns	ns	ns	ns	ns
Neisseraceae	0	0	0	5.7	ns	ns	ns	ns	ns	ns
Aeromonadaceae	3.2	0	0	0	ns	ns	ns	ns	ns	ns
Enterobacteriaceae	0	15.4	4.2	2.9	ns	ns	ns	ns	ns	ns
Morganellaceae	0	7.7	12.5	14.3	ns	ns	ns	ns	ns	ns
Pseudomonadaceae	12.9	0	0	0	ns	ns	ns	ns	ns	ns
Weeksellaceae	3.2	0	0	0	ns	ns	ns	ns	ns	ns

Abbreviations: A = autumn; Sp = spring; Sm = summer; W = winter; ns = not significant.

**Table 6:** Relative abundance (%) of bacteria (family level) according to age and reproductive state.

Bacterial Families	I < 2 yrs	I > 2 yrs	M < 2 yrs	M > 2 yrs	P < 2 yrs	P > 2 yrs
Brucellaceae	0	0	0	1.9	0	0
Rhizobiaceae	0	0	0	13.5	10	0
Aerococcaceae	14.3	0.0	0.0	0	0	0
Bacillaceae	42.9	20.0	0.0	5.8	5	3.3
Enterococcaceae	0	0	0	0	0	0
Micrococcaceae	0	0	0	1.9	0	36.7
Staphylococcaceae	28.6	60.0	66.7	50	45	36.7
Streptococcaceae	0	0	0	5.8	5	3.3
Burkholderiaceae	0	0	0	3.8	5	0
Neisseraceae	0	0	0	0	5	3.3
Aeromonadaceae	0	0	0	1.9	0	0
Enterobascteriaceae	14.3	0	0	3.8	5.0	3.3
Morganellaceae	0	20	0	5.8	15	13.3
Pseudomonadaceae	0	0	33.3	3.8	5	0
Weeksellaceae	0	0	0	1.9	0	0

Abbreviations: I = intact; M = multiparous; P = primiparous; yrs = years.

## Data Availability

Data are contained within the article.

## Supplementary Materials

The following supporting information can be downloaded at: https://www.mdpi.com/article/10.3390/ani14101501/s1, Table S1: Co-isolations of bacteria.
