# Supplementary material for: Aerobic Vaginal Microflora in Gestational and Non-Gestational Bitches (Canis lupus familiaris)
Source: Animals (Basel). 2024 May 18;14(10):1501. doi: 10.3390/ani14101501 (PMC11117226; doi:10.3390/ani14101501)
Supplement: Supplementary file 1 [file animals-14-01501-s001.zip › animals-2942141-supplementary.pdf]

Table S1. Co-isolations of bacteria

| G+ bacterias | | G- bacterias | |
|---|---|---|---|
| ***Staphylococcus β hemolytic*** | *Bacillus spp* | *E. coli* | |
| ***E. faecium*** | | *E. coli* | |
| ***S. xylosus*** | | *A. radiobacter* | *P. aeruginosa* |
| | | *A. radiobacter* | *O. anthropi* |
| ***Staphylococcus spp*** | | *A. radiobacter* | *A. hydrophila* |
| ***Staphylococcus spp*** | | *A. radiobacter* | |
| | | *A. radiobacter* | *P. aeruginosa* |
| ***Staphylococcus spp*** | | *A. radiobacter* | |
| ***Staphylococcus spp*** | | *A. radiobacter* | |
| ***Staphylococcus spp*** | | *P. aeruginosa* | |
| ***Staphylococcus spp*** | *Bacillus spp* | *Proteus spp* | |
| ***Staphylococcus α hemolytic*** | | *Proteus spp* | |
| ***Staphylococcus β hemolytic*** | | *P. mirabilis* | *C. violaceum* |
| ***A. viridans*** | *Bacillus spp* | | |
| ***M. luteus*** | *Streptococcus spp* | | |
| ***Staphylococcus spp*** | | *Klebsiella spp* | |
| ***S. anginosus/sanguinis*** | *Bacillus spp* | | |
| ***Staphylococcus spp*** | | | |
| | *Bacillus spp* | *E. coli* | |
| ***Staphylococcus β hemolytic*** | | | |
| | | *Proteus spp* | *P. vulgaris* |
| ***Staphylococcus spp*** | *Streptococcus α hemolitic* | | |
| ***Staphylococcus spp*** | | | *B.mallei* |
| ***Staphylococcus β hemolytic*** | *Bacillus spp* | | |
| ***Staphylococcus spp*** | *B. pumilus* | | |
| | *Bacillus spp* | *Proteus spp* | *Klebsiella spp* |

Abbreviations: *A. viridans*, *Aerococcus viridans*; *A. hydrophila*, *Aeromonas hydrophila*; *A. radiobacter*, *Agrobacterium radiobacter*; *B. pumilus*, *Bacillus pumillus*; *B. mallei*, *Burkholderia mallei*; *C. violaceum*, *Chromobacterium violaceum*; *E. coli*, *Escherichia coli*; *E. faecium*, *Enterococcus faecium*; *M. luteus*, *Micrococcus luteus*; *O. anthropic*, *Ochrobactrum anthropi*; *P. vulgaris*, *Proteus vulgaris*; *P. aeruginosa*, *Pseudomonas aeruginosa*; *S. xylosus*, *Staphylococcus xylosus*; *S. anginosus*, *Streptococcus anginosus*; *S. sanguinis*, *Streptococcus sanguinis*.
